# Kynurenine/Tryptophan Ratio as a Potential Blood-Based Biomarker in Non-Small Cell Lung Cancer

**DOI:** 10.3390/ijms22094403

**Published:** 2021-04-22

**Authors:** Martina Mandarano, Elena Orecchini, Guido Bellezza, Jacopo Vannucci, Vienna Ludovini, Sara Baglivo, Francesca Romana Tofanetti, Rita Chiari, Elisabetta Loreti, Francesco Puma, Angelo Sidoni, Maria Laura Belladonna

**Affiliations:** 1 Section of Anatomic Pathology and Histology, Department of Medicine and Surgery, University of Perugia, 06129 Perugia, Italy; martina.mandarano@unipg.it (M.M.); guido.bellezza@unipg.it (G.B.); elisabetta.loreti@unipg.it (E.L.); angelo.sidoni@unipg.it (A.S.); 2 Section of Pharmacology, Department of Medicine and Surgery, University of Perugia, 06129 Perugia, Italy; elena.orecchini@gmail.com; 3 Section of Thoracic Surgery, Department of Medicine and Surgery, University of Perugia, 06129 Perugia, Italy; jacopo.vannucci@uniroma1.it (J.V.); francesco.puma@unipg.it (F.P.); 4 Medical Oncology Division, Santa Maria della Misericordia Hospital, 06132 Perugia, Italy; vienna.ludovini@ospedale.perugia.it (V.L.); sara.baglivo@ospedale.perugia.it (S.B.); francesca.tofanetti@ospedale.perugia.it (F.R.T.); 5 Division of Medical Oncology, Ospedali Riuniti Padova Sud, 35043 Monselice, Italy; rita.chiari@aulss6.veneto.it

**Keywords:** kynurenine/tryptophan (Kyn/Trp) ratio, indoleamine-2,3-dioxygenase 1 (IDO1), non-small cell lung cancer (NSCLC), immunohistochemical biomarkers, serum biomarkers

## Abstract

The enzyme indoleamine 2,3-dioxygenase 1 (IDO1) degrade tryptophan (Trp) into kynurenine (Kyn) at the initial step of an enzymatic pathway affecting T cell proliferation. IDO1 is highly expressed in various cancer types and associated with poor prognosis. Nevertheless, the serum Kyn/Trp concentration ratio has been suggested as a marker of cancer-associated immune suppression. We measured Kyn and Trp in blood samples of a wide cohort of non-small-cell lung cancer (NSCLC) patients, before they underwent surgery, and analyzed possible correlations of the Kyn/Trp ratio with either IDO1 expression or clinical–pathological parameters. Low Kyn/Trp significantly correlated with low IDO1 expression and never-smoker patients; while high Kyn/Trp was significantly associated with older (≥68 years) patients, advanced tumor stage, and squamous cell carcinoma (Sqcc), rather than the adenocarcinoma (Adc) histotype. Moreover, high Kyn/Trp was associated, among the Adc group, with higher tumor stages (II and III), and, among the Sqcc group, with a high density of tumor-infiltrating lymphocytes. A trend correlating the high Kyn/Trp ratio with the probability of recurrences from NSCLC was also found. In conclusion, high serum Kyn/Trp ratio, associated with clinical and histopathological parameters, may serve as a serum biomarker to optimize risk stratification and therapy of NSCLC patients.

## 1. Introduction

Lung cancer is the main cause of cancer-related death worldwide [1]. About 80% of the disease is classified into non-small-cell lung cancer (NSCLC). Among them, approximately 20% of cases are localized disease (stages I and II), 25% are locally advanced disease (stage III), and 55% of cases are metastatic disease (stage IV) [2]. Surgical resection is the preferred treatment option for patients with early-stage and some locally advanced NSCLC. Genomic profiling of resected tumors, allowing identification of potentially targetable alterations and/or eligibility for immune checkpoint inhibition, is now included in standard care and is considered an essential diagnostic tool to improve clinical outcomes in particular patient subgroups [3,4]. Biomarkers analyzed in such surgical samples, or traditional biopsy (particularly invasive in NSCLC patients), reflect tumor and disease status at a specific time; that of tissue removal. Nevertheless, to monitor tumor evolution over time, the dosage of cancer-associated markers in liquid biopsy is now considered an attractive means for the optimization of cancer therapeutic protocols. In a liquid biopsy, circulating tumor cells and cell-free DNA are principally investigated to improve lung cancer screening, detect minimal residual disease, monitor systemic treatment initiation and response, and determine the development of resistance [5]. However, metabolites of the kynurenine (Kyn) pathway also appear to be interesting candidates to be dosed in the blood to follow cancer evolution and control it by therapeutic intervention.

Kyn pathway, a tryptophan (Trp)-degrading enzymatic cascade controlled at the first rate-limiting step by indoleamine-2,3-dioxygenase 1 (IDO1), has been associated with a poorer prognosis in cancer patients [6,7,8]. IDO1 expression/activity has been observed in tumor cells as well as in the tumor-surrounding stroma, which is composed of endothelial cells, immune cells, fibroblasts, and mesenchymal cells [9]. Enhanced Trp breakdown occurring in the tumor microenvironment, reflected by decreased Trp and elevated Kyn concentrations in the peripheral blood, is often observed in cancer patients and related to tumor progression and poor clinical outcome [10]. In cancer immunoediting, IDO1 induction in malignant cells and their microenvironment is a key mechanism to modulate anti-tumor immune responses and thus escape immune surveillance [11,12]. Decreased Trp availability and accumulation of Trp metabolites, such as the kynurenines, directly affect immune T cell proliferation and functions, and induce their apoptosis [13,14]. Based on these mechanisms, IDO1 expression in tumor sites and circulating Trp and Kyn (evaluated as the Kyn/Trp ratio) have been hypothesized as possible markers of cancer progression [15,16]. In particular, recent studies have demonstrated that the Kyn/Trp ratio is a promising surveillance biomarker for several cancer types. Plasma and urinary Kyn/Trp ratio are higher in patients with bladder cancer [17]. In advanced melanoma and renal cell carcinoma patients treated with nivolumab, the serum Kyn/Trp ratio increases as an adaptive resistance mechanism associated with worse overall survival [18]. In serum of glioblastoma multiforme patients, the Kyn/Trp index seems to be a relevant clinical benchmark, providing prognostic value for enrolment in immunotherapeutic regimens [19]. In NSCLC patients with solid tumors treated with immunotherapy, both prognostic and predictive values of serum Kyn/Trp ratio have been proposed [20].

Implementing the use of non-invasive biomarkers (i.e., measured by liquid biopsy) is a relevant research target for both prognostic and therapeutic purposes. In the present study, we evaluated the Kyn/Trp ratio dosed in blood samples of a wide cohort of NSCLC resected patients and aimed to correlate this value with several clinical–pathological parameters and to patients’ overall survival (OS) and disease-free survival (DFS), to provide new insights contributing to define the Kyn/Trp ratio as a useful serum biomarker for better risk stratification and therapy improvement of NSCLC patients.

## 2. Results

### 2.1. Patients Series

One hundred and eighty patients were eligible for the study. Patients were all Caucasian; the median age was 68 years (range 38–84) with a median follow-up period of 51 months (range 1–107 months). One hundred and twenty-seven (71%) patients were males; most of the patients were either current smokers or former smokers (164; 91%). Sixty-nine (38%) patients relapsed after surgery and fifty-seven (32%) died from NSCLC.

### 2.2. Histopathological Findings

Surgical specimens from radically resected tumors were analyzed by hematoxylin and eosin (H&E) staining for histopathological determination. One hundred and eighteen (66%) patients suffered from adenocarcinoma (Table 1), which often presented either a lepidic, acinar, papillary, or mucinous primary pattern (92; 78%, data not shown), less aggressive than solid pattern. The pathological stage of the disease was determined according to the classification of the 8th Edition for Cancer Staging by the American Joint Committee on Cancer (AJCC). Stage I was the most frequent (117; 65%). The majority of adenocarcinomas belonged to the stage I group (73; 62%), whereas most of the squamous cell carcinomas were in the stage II–III group (36; 58%).

### 2.3. Analysis of Tumor-Infiltrating Lymphocytes (TILs)

**Figure 1:**
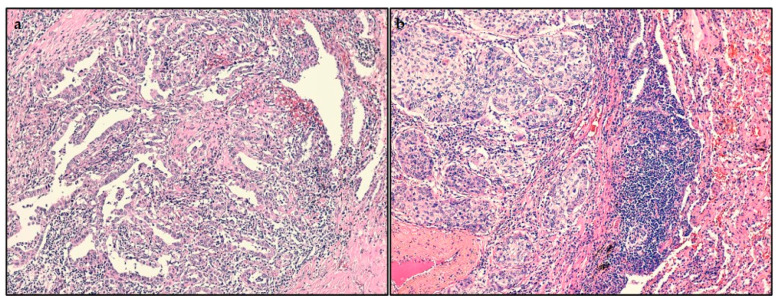
Histopathological analysis by hematoxylin and eosin (H&E) staining. (**a**) Adenocarcinoma of the lung, with a high intratumoral density of tumor-infiltrating lymphocytes (TILs), magnification: 100×; (**b**) squamous cell carcinoma of the lung, with a high mixed density of TILs, magnification: 100×.

Through H&E staining, we analyzed the localization of TILs in surgical specimens. More frequently, it was intratumoral (83; 46%), regarding the entire series and the adenocarcinomas group (63; 53%) (Figure 1a). On the other hand, the mixed localization was the most observed among the squamous cell carcinomas (34; 55%) (Figure 1b).

### 2.4. Immunohistochemical Analysis of IDO1 and Programmed Cell Death Ligand-1 (PD-L1)

**Figure 2:**
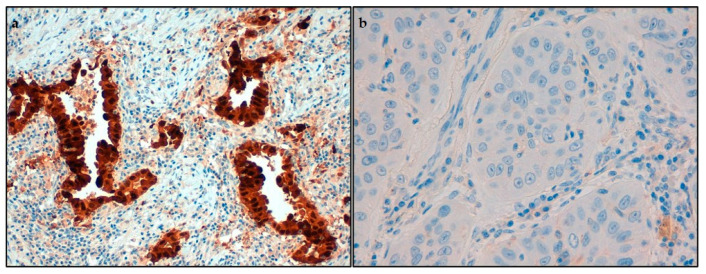
Immunohistochemical analysis of indoleamine-2,3-dioxygenase 1 (IDO1) and programmed cell death ligand-1 (PD-L1) expression. (**a**) Immunohistochemical high expression of IDO1 in adenocarcinoma; (**b**) immunohistochemical low expression of PD-L1 in squamous cell carcinoma. Original magnification: (**a**) 200×, (**b**) 400×.

Expression of IDO1, the tolerogenic enzyme converting Trp into Kyn, was investigated on neoplastic cells by immunohistochemical analysis. We found that most of the tumors presented a high expression of IDO1 molecule (101; 56%, Figure 2a), both among adenocarcinomas (65; 55%) and squamous cell carcinomas (36; 58%). On neoplastic cells, we also evaluated the immunostaining for PD-L1, a ligand inhibiting T cell proliferation and associated with increased tumor aggressiveness. With a trend opposite to IDO1, many of the neoplasms presented a low expression of PD-L1 (139; 77%, Figure 2b), both regarding adenocarcinomas (97; 82%) and squamous cell carcinomas (42; 68%).

### 2.5. Kyn/Trp Ratio and Clinical Parameters Associations

**Figure 3:**
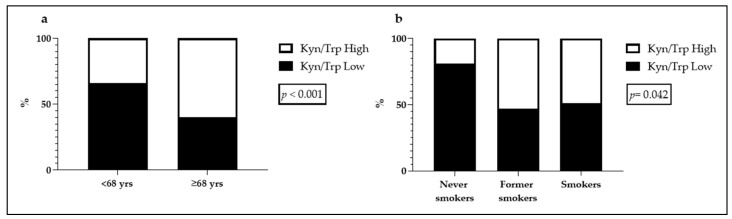
Statistically significant associations between kynurenine/tryptophan (Kyn/Trp) ratio and clinical parameters. (**a**) Association between Kyn/Trp ratio and patients’ age; (**b**) association between Kyn/Trp ratio and smoking status.

Before the NSCLC patients underwent surgery, blood samples were collected for following HPLC analysis of Kyn and Trp relative concentration. Two groups of the Kyn/Trp ratio (low and high) were used for the subsequent evaluation of their associations with clinical–pathological parameters. The Kyn/Trp Low group encompassed the Kyn/Trp ratio values equal to or below the median value of 58, while the Kyn/Trp High group included the values above 58. If the serum concentration of Kyn was undetectable, the corresponding patient was excluded from the analyses. The associations between Kyn/Trp ratio and the clinical parameters are shown in Table 1. The analysis revealed that just over half of the patients presented a low Kyn/Trp ratio (93; 52%) and that Kyn/Trp ratio was higher in over 68-year-old patients (*p* < 0.001). On the other hand, there was a low Kyn/Trp ratio in patients who were never smokers (*p* = 0.042). The statistically significant associations found are summarized in Figure 3.

### 2.6. Kyn/Trp Ratio and Histopathological Findings Associations

Via HPLC, we analyzed Kyn and Trp serum concentration. We found values between 1.0240 and 30.3154 µM for Kyn (mean = 3.9772 ± 2.4368 µM; median = 3.6952 µM) and between 13.0968 and 592.2520 µM for Trp (mean = 65.0871 ± 43.4785 µM; median = 62.0500 µM).

**Figure 4:**
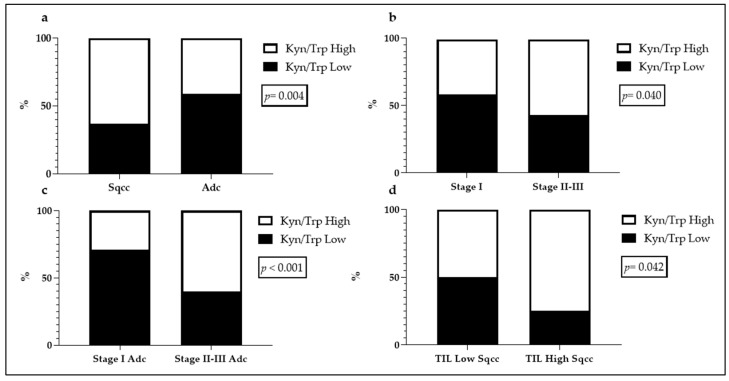
Statistically significant associations between Kyn/Trp ratio and histopathological findings. (**a**) Association between Kyn/Trp and histotype; (**b**) association between Kyn/Trp and stage of disease; (**c**) association between Kyn/Trp and stage of disease, adenocarcinomas (Adc) group; (**d**) association between Kyn/Trp and TILs density, squamous cell carcinomas (Sqcc) group.

We investigated possible correlations between Kyn/Trp ratio and the histopathological findings and summarized the analyzed associations in Table 2. A high Kyn/Trp ratio was found among squamous cell carcinoma histotype (*p* = 0.004) and, among this group, when the tumor presented a high TIL density (*p* = 0.042; Figure 4). Moreover, the higher the Kyn/Trp, the higher the stage of adenocarcinomas (*p* < 0.001; Figure 4).

### 2.7. Kyn/Trp Ratio and Immunohistochemical Associations

**Figure 5:**
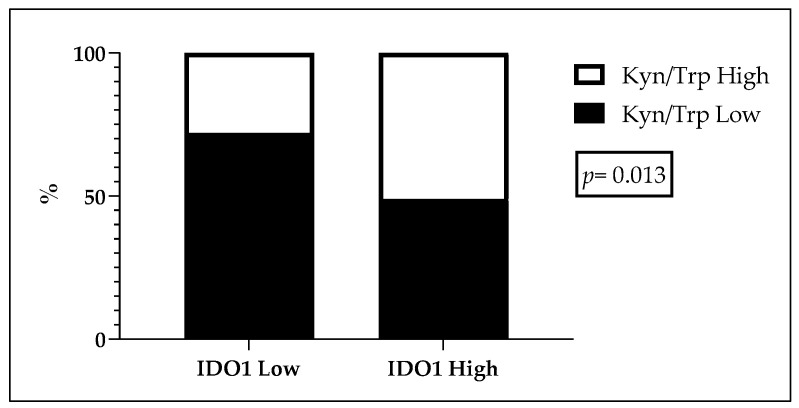
Statistically significant association between low Kyn/Trp ratio and low immunohistochemical expression of IDO1, adenocarcinomas group.

The immunohistochemical analysis included the evaluation of either IDO1 or PD-L1 protein expressed by tumor cells in surgical specimens. For both IDO1 and PD-L1, low and high expression groups were identified according to a score assignment procedure, previously published [21] and here described in the Material and Methods. The only statistically significant association we found was between a low Kyn/Trp ratio and a low IDO1 expression in the adenocarcinoma group (*p* = 0.013; Table 3 and Figure 5).

We found no further statistically significant associations between Kyn/Trp ratio and the clinical–pathological parameters among those we explored (Table 1, Table 2 and Table 3).

### 2.8. Kyn/Trp Ratio and Survivals Analyses

We conducted survival analyses in the whole cohort, in the subgroups of patients having adenocarcinoma or squamous cell carcinoma, and in the subgroups at stages II–III of disease. To investigate a possible prognostic value of the serum Kyn/Trp ratio, within each of the four groups, we compared OS and DFS curves of Kyn/Trp low vs. high groups. In the heterogeneous group of the whole NSCLC series, as in both the carcinoma histotype and advanced stage subgroups, OS analysis gave no statistically significant results, since the two groups of Kyn/Trp low and high were similarly distributed among the patients who died or survived during the follow-up period (Table 4). Similarly, among the two Kyn/Trp low and high groups, we did not find any statistically significant result by the DFS analysis on the patients who had a relapse and who did not have a recurrence from NSCLC (Table 4).

**Figure 6:**
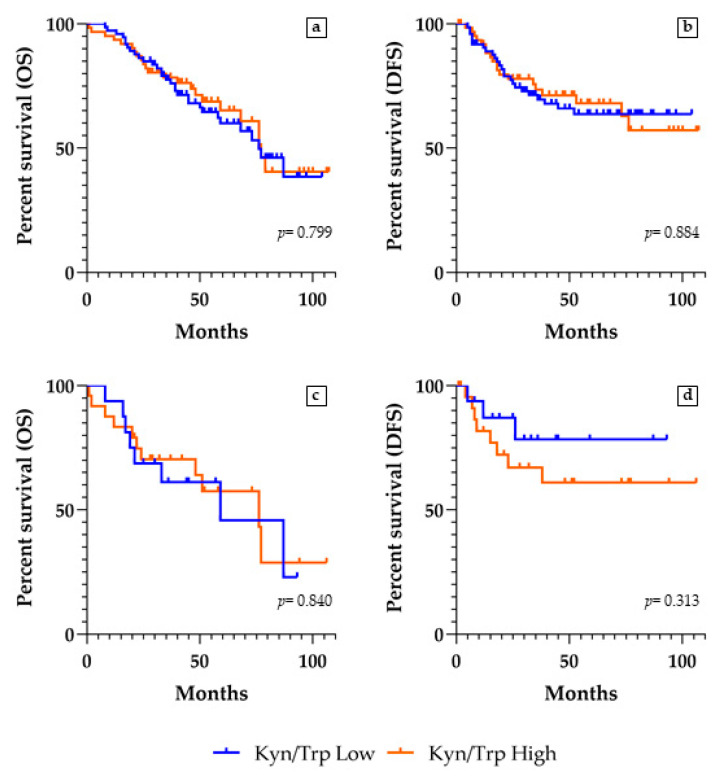
Overall survival (OS) and disease-free survival (DFS) curves according to the Kyn/Trp ratio. (**a**) OS and (**b**) DFS analysis of patients who did not undergo adjuvant therapies; (**c**) OS and (**d**) DFS analysis of patients in stages II–III of disease who did not undergo adjuvant therapies.

However, in a selected population of stage II–III patients who did not undergo adjuvant therapies, the DFS analysis showed a clear trend, even though not statistically significant (*p* = 0.313, HR 1.95, 95% CI 0.59–6.46), suggesting that the high Kyn/Trp ratio could correlate with a worse prognosis (Table 5 and Figure 6).

## 3. Discussion

Many studies demonstrate that solid tumors may escape immunosurveillance through upregulation of the IDO1 enzyme in cancer cells and tumor microenvironment, a condition associated with poor clinical outcomes [9,16]. IDO1 increased expression is reflected by the relative concentration of Kyn compared to Trp; hence, the Kyn/Trp ratio can be used as a prognostic clinicopathological marker to monitor cancer invasiveness and progression [22]. The involvement of IDO1 activity in cancer immunoediting and growth seems to be so relevant that targeting this enzyme represents an attractive approach for cancer immunotherapeutic intervention [23]. The inhibition of IDO1 catalytic activity is currently under investigation in several clinical trials and at least eight small molecule IDO1 inhibitors are evaluated as possible new anti-cancer drugs [24]. Lacking an assay allowing us to directly determine the activity of IDO1 enzyme in tumor tissues, most of the preclinical data provided by these IDO1-inhibition trials were derived from serum concentrations of Trp, Kyn, or the Kyn/Trp ratio, chosen as surrogate indicators for IDO1 activity evaluation [25,26,27,28]. Based on previous literature and to enlighten its effectiveness as a serum tumor biomarker, we looked for statistical associations between Kyn/Trp concentration ratio and several clinical, histopathological, and immunohistochemical parameters in a cohort of NSCLC patients who underwent surgical resection of tumors.

In our study, we assigned patients to the high or low Kyn/Trp group, depending on whether their Kyn/Trp ratio was above or below the median value of 58, respectively. Evaluating patients ranging between 38 and 84 years of age, with a median value of 68 years old, we found a positive and statistically significant correlation between the high Kyn/Trp ratio condition and the over-68-year-old patients’ age. The Kyn/Trp ratio, measured in blood, is documented to be robustly associated with aging in humans [29]. The more inflammation derived from age-related tissue damage increases, the more Kyn pathway, depleting tryptophan and producing tolerogenic metabolites, potentiated as a homeostatic response to control the so-called “inflammaging”. Thus, the Kyn/Trp ratio met the criteria for a biological age biomarker [30]. In our study, the presence of tumor tissue and relative inflamed tumor microenvironment could be a possible trigger for further increase of Trp catabolism, substantiating our finding of a higher Kyn/Trp ratio in older patients.

The smoking parameter appeared to be not related to Trp catabolism in our analysis, although a decreased serum activity of the IDO1 enzyme had been documented in a study on a representative cohort of smoking subjects not affected by cancer [31]. However, since we found that never-smoker status was statistically associated with the low Kyn/Trp ratio, Trp catabolism could be hypothesized as a cancerogenic mechanism in smoker NSCLC patients. To confirm this hypothesis, more data relative to the poorly represented population of never-smoker lung cancer patients are needed.

Another intriguing finding regards the association between the high Kyn/Trp ratio and the squamous cell histotype of NSCLC in our series. This could reflect the already established concept, according to which many suppressor gene alterations are specific features of squamous cell carcinoma, with consequent chromosomal instability and accumulation of somatic mutations. The latter increase the production of neoantigens, which can abruptly activate the innate immune response [32]. The resulting inflammatory microenvironment may ensue the overexpression of IDO1 [21], and, consequently, the activation of the kynurenine pathway, which leads to an increase of Kyn/Trp ratio in this kind of NSCLC. Moreover, recent studies have associated the role of IDO1 in developing immunotherapy resistance in squamous cell carcinomas, independently from the tumor site [20]. The typical hyperactivation of the IDO1 pathway in an inflammatory microenvironment, which is usually characterized by increased release of IFNγ [33], could also account for the statistically significant association between a high Kyn/Trp ratio and a high density of TILs at the tumor site among the squamous cell carcinoma group of the current study. In fact, as above mentioned, this tumor histotype usually elicits a higher immune stimulation because of its intrinsic higher antigenicity [32]. Furthermore, this finding seems partially to confirm data obtained from our recent study [21], which demonstrated that squamous cell lung cancers with a high TIL density presented a concomitant higher IDO1 immunohistochemical expression.

A recent study about cervical cancer found a correlation between the advanced stage of the disease and the higher Kyn/Trp ratio [34], and other authors described a relationship between an elevated Kyn/Trp ratio and known biologically aggressive neoplasms, such as glioblastoma [35]. These results, together with our statistically significant association between high Kyn/Trp ratio and more advanced NSCLC stages, among both the whole cohort of patients and the adenocarcinoma group, suggests the activation of the Kyn pathway in the tumor microenvironment as linked to poor clinicopathological parameters and, consequently, to more aggressive neoplasms. Furthermore, we confirmed the observation of Wang et al. [36], according to which a high level of IDO1 activity, indirectly measured as Kyn/Trp ratio, is typical of adenocarcinoma advanced stages rather than non-adenocarcinoma histotype of NSCLCs. This could lead to set up a multidisciplinary diagnostic workflow that would allow the early histopathological identification of NSCLC patients with adenocarcinoma, to specifically determine their serum Kyn/Trp ratio and identify the lung cancers which could benefit from therapies targeting the IDO1-kynurenine pathway axis. This potential additional therapeutic target would not only delay the tumor progression in the early stages of NSCLC, but also support other chemotherapeutic agents for the more advanced—and often resistant to currently available therapies—stages of this cancer type. Nevertheless, further studies, particularly including adequate patients’ follow-up periods and subsequent survival analysis, are needed to confirm our hypothesis.

In the series we analyzed in the present study, there was no statistically significant association between a high Kyn/Trp ratio and high immunohistochemical expression of IDO1, neither in the whole NSCLC population nor within the two histotype subgroups. To explain why a high Kyn/Trp ratio did not correlate with a high histochemical level of IDO1, we can speculate that not only tumor tissue but also other tumor microenvironment cell types [9] can importantly contribute to the tryptophan catabolism attempting to exhaust innate immune system activation in NSCLC. On the other hand, the finding of a low immunohistochemical expression of IDO1, statistically associated with a low Kyn/Trp ratio among NSCLC adenocarcinomas group, corroborated the need for preliminary histopathological screening to identify patients going to serum Kyn/Trp determination, to distinguish those that really could benefit from a blockade of Kyn pathway. As a matter of fact, we analyzed the immunohistochemical expression of IDO1 only at the tumor cell level, but the Kyn/Trp ratio could be the result of the catalytic enzyme overexpression in other cells of the tumor microenvironment, such as the dendritic cells [36]. Therefore, a better cellular stratification in NSCLCs microenvironment, particularly among adenocarcinomas, is needed in these patients.

Because of its relevance in triggering cancer-related tolerance in NSCLC and for its targetability in immunotherapy with immune-checkpoint inhibitors, which NSCLC is particularly responsive to [37], we chose to also investigate the immunohistochemical expression of the immune-checkpoint molecule PD-L1. The absence of statistically significant associations between a high or low Kyn/Trp ratio, and high or low immunohistochemical expression of PD-L1, in the whole NSCLC population, or within the two histotype subgroups, suggests that these two markers should be carefully considered in the stratification of patients to be treated with an immunotherapy combining the inhibition of IDO1 and the blockade of programmed cell death-1 (PD-1)/PD-L1 interaction. Such dual-agent therapy has been proposed for NSCLC [38,39] and is currently evaluated by oncological clinical trials [40] to maximize the clinical benefit of immune-checkpoint inhibitors, not effective on a significant fraction of NSCLC patients [37]. In evaluating the criteria for stratification of patients, not only the immunohistochemical expression of PD-L1 in tumor cells should be considered, but also the circulating component of soluble PD-L1, contributing to PD-1 receptor activation and, consequently, to the onset of cancer-permissive tolerance [41,42]. Thus, the absence of correlations between the serum Kyn/Trp ratio and PD-L1 immunohistochemical expression might suggest the importance of an integrated evaluation of histological (PD-L1 and IDO1) and serum (soluble PD-L1 and Kyn/Trp ratio) markers for specifically targeted NSCLC immunotherapy.

Though the Kyn/Trp ratio has been proposed as a useful prognostic clinicopathological marker [19,20,22], many aspects concerning its survival predictive value remain elusive. In our study, we verified that the Kyn/Trp ratio evaluated at the pre-surgical time in heterogeneous populations of NSCLC does not allow correlations between the Kyn/Trp serum parameter and patients’ OS or DFS to be defined. In such populations, different factors could have influenced the exitus occurrence, including the administration of adjuvant therapy, or the absence of it. Restricting the analysis to a subgroup of stage II–III NSCLC patients not treated with adjuvant therapy, a dichotomic trend can be enlightened, suggesting that a correlation between the high Kyn/Trp ratio and a poor prognosis could be established more successfully in the more advanced than in the early stages of the disease. This finding, obtained in our small subgroup of stage II–III NSCLC patients who did not undergo adjuvant therapy, needs to be statistically confirmed by further studies on a larger population. However, besides its possible use as a prognostic parameter in advanced-stage patients, we believe in the potentialities of the serum Kyn/Trp ratio as a non-invasive biomarker in the follow-up of NSCLC to monitor over time the level of IDO1 activity and its impact on immune responses, and to choose a new IDO1-based target therapy in addition to the currently available ones. Further investigations aimed to evaluate the modifications of the Kyn/Trp ratio in combination with other serum biomarkers would optimize the prognostic risk stratification of NSCLC patients.

## 4. Materials and Methods

### 4.1. Patients’ Recruitment

The current study was approved by the local ethics committee of Comitato Etico delle Aziende Sanitarie della Regione Umbria CEAS Umbria (15 November 2013), which assigned the protocol code N. 2216/13 to the present research. Patients were recruited from 2009 to 2015 at S. M. Misericordia Hospital, Perugia, Italy, selecting all the NSCLC cases, which underwent surgery with curative intent. Moreover, only the cases with both known clinical parameters were selected (Table 1).

### 4.2. Histopathological and Immunohistochemical Determinations

At the section of Anatomic Pathology and Histology, Department of Medicine and Surgery, University of Perugia, the surgical specimens were formalin-fixed (10% buffered formalin) and paraffin-embedded (FFPE). Sections of 4 μm were performed and placed on slides with a permanent positive charged surface for both H&E and immunohistochemical (IHC) stains. The H&E stain was carried out using a Leica ST5020 Multistainer (Leica Biosystems, Nußloch, Germany), using the kit ST Infinity H&E Staining System (Leica Biosystems, Richmond, IL, USA). The BOND-III fully automated immunohistochemistry stainer (LeicaBiosystems, Nußloch, Germany) performed the IHC stains (peroxidase immunoenzymatic reaction with development in diaminobenzidine). Proper positive and negative controls were included. A histological subtype was assigned according to 2015 World Health Organization (WHO) classification for lung tumors. Only tumors classified as adenocarcinomas or as squamous cell carcinomas were considered for the study.

The pathological stage was assigned according to the 8th Edition for Cancer Staging by the AJCC. According to this staging of disease, the NSCLCs were grouped into a stage I group, including stages from IA1 to IB, and a stage II–III group, encompassing the stages from IIA to IIIB.

The localization of TILs (absent; intratumoral = among tumor cells; peritumoral = at the interface between the neoplasia and healthy lung parenchyma; mixed = mixture of the last two localizations) and the density of TILs, according to the percentage of lymphocytes observed in a given localization (low < 20%; high ≥ 20%) were determined by experienced pathologists (G. Bellezza, M. Mandarano, and A. Sidoni) using H&E slides, as previously reported [21]. The same pathologists analyzed the immunohistochemical expression, on neoplastic cells, of IDO1 (courtesy of Professor Benoit J Van den Eynde, Ludwing Institute for Cancer Research, Louvain-la-Neuve, Belgium, clone 4.16H1 [43]; dilution 1:1000) and PD-L1 (Cell Signaling Technology, Danvers, MA, USA, Cat# 13684S, RRID:AB_2687655, dilution 1:200), as previously reported [21], obtaining an H score resulting from the sum of the intensity of the stain (evaluated as 0: Absent; 1+: Mild; 2+: Moderate; 3+: Intense) and the percentage of the tumor cells labeled (0:0%; 1: 1–25%; 2: 26–50%; 3: 51–75%; 4: 76–100%). Thereafter, two groups of staining were considered for the study: A low expression one—scores from 0 to 2—and a high expression one—scores from 3 to 7.

### 4.3. Determination of Kyn and Trp Serum Concentration

Blood samples were collected from patients before they underwent surgery and immediately centrifuged to obtain serum samples. After deproteinization, Kyn and Trp concentration was measured by HPLC. Briefly, a Perkin Elmer (series 200 HPLC) instrument and a Kinetex C18 column (250 × 4.6 mm, 5 μm, 100 Å, Phenomenex, Torrance, CA, USA) were used for the analysis. The column was maintained at a temperature of 25 °C and a pressure of 1800 PSI. Samples were eluted with a phase containing 10 mM NaH_2_PO_4_ (pH = 3.0; 99%) and methanol (1%) (Sigma–Aldrich, St. Louis, MO, USA), with a flow rate of 1.3 mL/min. Kyn was detected at 360 nm and Trp was detected at 220 nm by a UV detector. The software TURBOCHROM 4 was used for evaluating the concentration of Kyn and Trp in samples by mean of a calibration curve. Two groups of Kyn/Trp ratio were used for the subsequent evaluations of their associations with clinical–pathological parameters: The Kyn/Trp low, encompassing the value of the ratio ≤ 58, and the Kyn/Trp high, which included the value of ratio > 58. When the value of Kyn was undetectable, the corresponding patient was excluded from the analyses.

### 4.4. Statistical Analysis

The statistical analysis was performed using GraphPad Prism 8 (GraphPad Software Inc., San Diego, CA, USA). Categorical variables were presented as frequencies with row and column percentages. Categorical variables were compared between the groups using Chi-square test or Fisher’s exact test as appropriate. OS and DFS univariate analyses were performed for the subgroups determined by the Kyn/Trp ratio using a log-rank test and were represented by Kaplan–Meier curves. *p*-values (*p*) less than 0.05 were considered statistically significant.

## 5. Conclusions

In our study on resected NSCLC tumors, among other correlations, we found that the high Kyn/Trp ratio was significantly associated with the more aggressive squamous cell histotype and a more advanced tumor stage. These findings, confirming previous observations reported in several cancer types, importantly suggest the value of the non-invasive analysis of the serum Kyn/Trp ratio as a follow-up marker contributing to the indication of cancer progression. However, the serum determination of the Kyn/Trp ratio would be inadequate if used alone for the risk stratification of NSCLC patients and the identification of the best-personalized therapy. A more complex and complementary model of tumor biomarker analysis, both at histopathological and molecular/serum levels, should be considered for the best patients’ risk stratification and therapy.

## Figures and Tables

**Table 1:** Serum Kyn/Trp ratio and clinical parameters associations.

Parameter	Kyn/Trp Low		Kyn/Trp High		*p* Value	Total	
	N	%	N	%		N	%
	**93**	**52**	**87**	**48**		**180**	**100**
**Gender**							
Male	62	49	65	51	0.237	127	71
Female	31	58	22	42		53	29
**Age**							
<68 years	54	66	28	34	<0.001	82	46
≥68 years	39	40	59	60		98	54
**Smokers**							
Current	38	57	37	49	0.042	75	42
Former	42	47	47	53		89	49
Never	13	81	3	19		16	9

**Table 2:** Serum Kyn/Trp ratio and histopathological findings associations.

Parameter	Kyn/Trp Low		Kyn/Trp High		*p* Value	Total	
	N	%	N	%		N	%
	**93**	**52**	**87**	**48**		**180**	**100**
**Histotype**							
Adc ^1^	70	59	48	41	0.004	118	66
Sqcc ^2^	23	37	39	63		62	34
**Stage**							
I	58	59	41	41	0.040	99	55
II–III	35	43	46	57		81	45
Adc ^1^ stage						118	100
I	52	71	21	29	<0.001	73	62
II–III	18	40	27	60		45	38
Sqcc ^2^ stage						62	100
I	6	23	20	77	0.066	26	42
II–III	17	47	19	53		36	58
**TILs Density**							
Low	50	56	40	44	0.296	90	50
High	43	48	47	52		90	50
Adc ^1^						118	100
Low	35	58	25	42	0.824	60	51
High	35	60	23	40		58	49
Sqcc ^2^						62	100
Low	15	50	15	50	0.042	30	48
High	8	25	24	75		32	52
**TILs Localization**							
Intratumoral	45	54	58	46	0.418	83	46
Peritumoral	4	31	9	69		13	7
Mixed	42	53	37	47		79	44
Absent	2	40	3	60		5	3
Adc ^1^						118	100
Intratumoral	37	59	26	41	0.446	63	53
Peritumoral	3	50	3	50		6	5
Mixed	29	64	16	36		45	38
Absent	1	25	3	75		4	4
Sqcc ^2^						62	100
Intratumoral	8	40	12	60	0.341	20	32
Peritumoral	1	14	6	86		7	11
Mixed	18	38	21	62		34	55
Absent	1	100	0	0		1	2

^1^ Adc: Adenocarcinoma. ^2^ Sqcc: Squamous cell carcinoma.

**Table 3:** Serum Kyn/Trp ratio and immunohistochemical findings associations.

Parameter	Kyn/Trp Low		Kyn/Trp High		*p* Value	Total	
	N	%	N	%		N	%
	**93**	**52**	**87**	**48**		**180**	**100**
**IDO1**							
Low	47	59	32	41	0.063	79	44
High	46	46	55	54		101	56
Adc ^1^						118	100
Low	38	72	15	28	0.013	53	45
High	32	49	33	51		65	55
Sqcc ^2^						62	100
Low	9	35	17	65	0.731	26	42
High	14	39	22	61		36	58
**PD-L1**							
Low	73	53	66	47	0.674	139	77
High	20	49	21	51		41	23
Adc ^1^						118	100
Low	60	62	37	38	0.229	97	82
High	10	48	11	52		21	18
Sqcc ^2^						62	100
Low	13	31	29	69	0.147	42	68
High	10	50	10	50		20	32

^1^ Adc: Adenocarcinoma. ^2^ Sqcc: Squamous cell carcinoma.

**Table 4:** Survival analyses according to serum Kyn/Trp ratio, whole cohort of NSCLC patients.

Kyn/Trp	Exitus (%)		*p* Value	HR ^1^ (95% CI ^2^)	Relapse (%)		*p* Value	HR ^1^ (95% CI ^2^)
	Yes	No			Yes	No		
**NSCLCs**								
Low	39(51)	54(52)	0.941	1.02 (0.65–1.60)	36(52)	57(51)	0.967	0.99 (0.62–1.59)
High	37(49)	50(48)			33(48)	5(49)		
**Adenocarcinomas**								
Low	30(60)	40(59)	0.595	1.16 (0.65–2.07)	31(58)	39(60)	0.701	1.11 (0.64–1.93)
High	20(40)	28(41)			22(42)	26(40)		
**Squamous cell carcinomas**								
Low	9(35)	14(39)	0.837	0.92 (0.41–2.09)	5(31)	18(39)	0.759	1.18 (0.42–3.29)
High	17(65)	22(61)			11(69)	28(61)		
**Stage II–III**								
Low	16(41)	19(45)	0.873	1.05 (0.56–1.99)	15(43)	20(43)	0.887	1.05 (0.54–2.05)
High	23(59)	23(55)			20(57)	26(57)		

^1^ HR: Hazard ratio; ^2^ CI: Confidence interval.

**Table 5:** Survival analyses according to serum Kyn/Trp ratio, subgroups of patients who did not undergo adjuvant therapy.

Kyn/Trp	Exitus (%)		*p* Value	HR ^1^ (95% CI ^2^)	Relapse (%)		*p* Value	HR ^1^ (95% CI ^2^)
	Yes	No			Yes	No		
**NSCLCs**								
Low	31(57)	42(52)	0.799	0.93 (0.55–1.60)	24(56)	49(53)	0.884	0.96 (0.52–1.74)
High	23(43)	39(48)			19(44)	43(47)		
**Adenocarcinomas**								
Low	25(69)	35(60)	0.905	0.96 (0.47–1.94)	23(64)	37(64)	0.859	1.06 (0.53–2.11)
High	11(31)	23(40)			13(36)	21(36)		
**Squamous cell carcinomas**								
Low	6(33)	7(30)	0.657	0.80 (0.29–2.23)	1(14)	12(35)	0.398	2.41 (0.47–12.39)
High	12(66)	16(70)			6(86)	22(65)		
**Stage II–III**								
Low	8(43)	8(38)	0.840	0.91 (0.36–2.28)	3(27)	13(45)	0.313	1.95 (0.59–6.46)
High	11(58)	13(62)			8(73)	16(55)		

^1^ HR: Hazard ratio; ^2^ CI: Confidence interval.

## Data Availability

The datasets analyzed in this article are not publicly available to respect the confidentiality and protection of patients’ data, in compliance with the processing of data covered and protected by the Italian Privacy Law and by the GDPR (General Data Protection Regulation, EU regulation no. 2016/679). However, requests for access to a properly anonymized dataset of the present article can be directed to M.M. (martina.mandarano@unipg.it).
